# The Use of Diagnosis-Related Group-Based Reimbursement in the Czech Hospital Care System

**DOI:** 10.3390/ijerph18105463

**Published:** 2021-05-20

**Authors:** Zuzana Kotherová, Martina Caithamlová, Juraj Nemec, Kateřina Dolejšová

**Affiliations:** 1 Department of Public and Social Policy, Institute of Sociological Studies, Faculty of Social Sciences, Charles University, Pekařská 16, 158 00 Praha 5, Czech Republic; 2 Department of Biomedical Technology, Faculty of Biomedical Engineering, Czech Technical University in Prague, nám. Sítná 3105, 272 01 Kladno 2, Czech Republic; martina.caithamlova@fbmi.cvut.cz (M.C.); Kata.dolejsova@gmail.com (K.D.); 3 Faculty of Economics and Administration, Masaryk University, Lipová 41a, 602 00 Brno, Czech Republic; juraj.nemec@umb.sk; 4 Faculty of Economics, Matej Bel University, Tajovského 10, 97401 Banská Bystrica, Slovakia

**Keywords:** DRG, Czech Republic, hospital economy

## Abstract

(1) Background: Diagnosis-Related Groups (DRG), one possibility of a hospital payment system, are currently used in most European countries. Introduced to the Czech system in the 1990s, the DRGs are currently used mainly for care reporting and partly for reimbursement. According to most experts, the use of DRG remain controversial. The goal of this paper was to study the effects of the current Czech DRG system on hospitals financing and, on this basis, to propose possible changes to the reimbursement mechanism in the Czech Republic. (2) Methods: Qualitative research methods were used for evaluating DRG mechanisms of application in three selected healthcare establishments in the CR in the period of 2012–2018. (3) Results: Our study shows that the current implementation of the DRG system is set up in a way that is very similar to traditional flat rates and is unlikely to yield major positive effects of the DRG mechanism, such as predictability of payments for hospitalisation cases, care quality and efficiency and transparent financing. (4) Conclusions: Based on our results, deep systemic change of the reimbursement mechanism in the Czech Republic is necessary. We propose five partial measures leading to the cultivation of the Czech DRG.

## 1. Introduction

Diagnosis-Related Groups (DRG) represent one possibility of a hospital payment system (see [1]). Their mechanism is based on the principle that all patients treated by a hospital are classified into a limited number of DRGs, which are supposed to be clinically meaningful and relatively homogenous in their resource consumption patterns [2,3,4]. Each DRG is associated with a specific cost weight or tariff, which is usually calculated from information about average treatment costs of patients falling within a specific DRG in a sample from other hospitals in the past.

DRG-based payments are currently used in most European countries, especially in acute hospital care (France, Germany, Austria, England, Sweden, Poland, Ireland, Estonia, Portugal, Spain, etc.). DRG systems are internationally used for three reasons: first, they should increase the transparency of services which are effectively provided in hospitals (that is, through patient classification, measuring hospital output, etc.); second, DRG-based payment systems should give incentives for the efficient use of resources within hospitals by paying hospitals on the basis of the number and type of cases treated; and finally, the combination of increased transparency and efficient use of resources is assumed to contribute to improving, or at least assuring, the level of quality of care [4]. On the other hand, DRGs may be connected to several bottlenecks, e.g., a lack of transparency, which tends to promote classification abuse and creates compromises between the quality of treatment and financial considerations [5,6].

The use of DRGs has long been one of the most controversial issues in the Czech healthcare community. Introduced to the Czech system in the 1990s after several attempts to increase its use, the DRG mechanism is currently used mainly for care reporting and partly for reimbursement. However, according to most experts, the working Czech system has created an environment where different healthcare providers are paid differently for the same services regardless of the quality of care, mostly due to historical peculiarities that have been preserved in the system, see for example [7]. Moreover, the Czech DRG system does not seem to motivate providers to higher efficiency. To improve the situation, a DRG Restart Project is currently underway in the Czech Republic aimed at improving the hospital care reimbursement mechanism; the first outcomes are expected to roll out in 2021, with the aim of reflecting the need to deliver evidence-based changes into the Czech hospital reimbursement system.

The goal of this paper is to study the effects of the current Czech DRG system on hospital financing and, on this basis, to propose possible changes to the reimbursement mechanism in the Czech Republic.

The paper is divided into three core parts. First, we provide the DRG literature review and present a background in financing of the Czech health service providers. Second, a case study is provided to illustrate how DRG works in the Czech Republic. Finally, in the light of our results, we discuss the situation and propose changes to the reimbursement mechanism in the Czech Republic in relation to changes proposed within the DRG Restart project.

## 2. DRG-Based Reimbursement on Hospital Level

In this section, the secondary literature review about DRG delivers an overview of the pros and cons of DRGs and their potentially expected effects on the behaviour of hospitals.

Traditionally, there have been several main forms of reimbursement methods used for financing the health service providers, see for example [8,9]. At the hospital level, most countries use retrospective forms, e.g., the fee-for-service reimbursement in which providers receive payment for each service rendered, or the episode-of-care reimbursement where providers receive a lump-sum for all the services they provide related to a condition or disease—such as DRG payments, for example.

The cultivation of DRG reimbursement payments might be seen as one of the priorities of many European reimbursement systems. Some countries created their own reimbursement system (e.g., England, The Netherlands) while others only transformed their already established systems (e.g., France, Germany, the Czech Republic, and Sweden). All systems have gone through frequent changes and case classification is widely different in different countries [10].

### 2.1. ‘Technical’ Problems of DRGs

The implementation of a DRG is connected with many problems and obstacles (a summary of the main issues is provided in Table 1).

There is a set of common issues connected to the effort to create a consistent definition of groups. First, the adequate number of groups needs to be tackled. It is possible to observe a rise in the number of groups, as in the German G-DRG where the number of groups sharply increased in 2005–2011 [5,10,11], as well as the tendence to decrease the number of groups (France, The Netherlands). The French experience shows that too many groups might be superfluous [12], and a similar trend was seen in the Netherlands (30,000 groups in 2010) when their number was reduced to 4400 in 2012 [10,13]. The second issue is connected to the number of classification levels. It seems that classification based on a cumulative score of secondary diagnoses provides a more accurate definition of case severity, taking into account patient polymorbidity [4,10,11,14]. This secondary diagnosis coding system increases the predictability of the reimbursement mechanism.

The intentional declaration of cases as more serious than they actually were might be seen as another shared point. The tendency to increase the seriousness of cases is a result of a poorly designed system that fails to take into account the severity of specific cases and does not allow hospitals to report the real cost. A solution may lie in a higher number of classification factors, which, however, would make the system more complicated and less straightforward for provider performance benchmarking. This approach has been taken by Sweden. In general, prioritising less severe cases or inequalities in their treatment depend on the level of competition among healthcare providers and is more prevalent among private healthcare providers (as can be seen in England and France). Some countries use age to clarify their classification, and age may even be applied differently in different areas of medical care. According to studies by Mason et al. and Geissler et al., half of 10 studied EU countries have DRG systems that use patient age as a factor in case classification for appendectomies but only two countries use age as a factor in unplanned hip replacements [15,16]. Once again, careful thought must be given to which cases warrant the use of patient age as a serious element for group classification. Patient age may also be taken into account in situations where the cumulative score of secondary diagnoses is used.

There is also a common tendency to incorporate quality indicators into the reimbursement system, and England may be seen as exceptional in this effort [10,13]. Of all the European systems, the English one gives the highest weight to quality and demonstrates how important it is to set clear rules and procedures for quality evaluation, although they do not necessarily lead to improvements in all areas of care [17]. Evaluation of care quality is closely linked to complication reporting. Some EU countries recognise not only the main and secondary diagnoses but also co-morbidities known at the time when a patient is admitted to hospital and complications that arise during hospitalisation, using markers to report these.

Base rate convergence is another common issue. Inspiration can be found in Germany, although the relative success and efficiency of two phases of base rate convergence in this system are debatable. The objective is to unify rates at the national level through a regulation corridor, but different base rates are defined for different federal states. A system of reference base rates and acceptable deviations (as a percentage) seems to be a logical approach to base rate convergence. The correct setting of the corridor is crucial because even in Germany, according to Klein-Hitpaß et al., many base rates were near the bottom of the agreed bracket [18].

Repeated hospitalisation and the length of stay are examples of classic DRG commonly solved problematic issues. Some countries, such as England and Germany, try to prevent repeat hospitalisations by not paying for the subsequent hospitalisation if it occurs within a certain period of time after the patient is released from the first hospitalisation. The effect of the length of hospitalisation on reimbursement per treatment day has been studied by Baron et al. in the French DRG, where the average reimbursement per treatment day depends on the total length of hospitalisation and defined standards [19]. Providers get paid more for shorter hospitalisations, however, above a certain number of treatment days the case must be coded under a greater severity level. The system also includes motivation for one-day hospitalisation in an orthopaedic ward with payments for a case without complications including overnight stay in the hospital being identical to payments for an out-patient procedure.

Ensuring professional coding represents another common issue. Setting correct coding details without putting too much burden on providers is of crucial importance. The impact of reporting quality on the amount of reimbursements was studied by Mieth et al. and it was discovered that higher revenues could be achieved in the Australian DRG system in up to 34% of cases through secondary diagnosis coding, but maximum profit was not correlated with the maximum number of diagnoses [20]. The accuracy of coding is also connected with the hospital staff’s professional approach as well as with the appropriate role of payers within the reimbursement system who, as mentioned by Jackson et al., should not be interested only in financial audits [11]. Simply creating an adequate classification, however, will not ensure correct coding of patients’ clinical states in DRG; assuring a longer term, pre- and post-graduate education for coders is essential.

Another common issue is connected with the decision about the type of cost management—obligatory (e.g., England or the Netherlands), voluntary or just recommended (Sweden). Managers of healthcare facilities cannot make decisions without having the reliable cost information. In order to measure the financial performance more accurately, the so-called Activity-Based Costing (ABC) method may be used. In the ABC calculation, hospital procedures are assigned to events that generate costs.

Finally, there is the question of the level of centralisation. Even if, in most systems, the state plays the role of guarantor of care quality, the French experience shows that healthcare providers may have greater autonomy, and reimbursement might be managed at the national level to a lesser degree [12].

**Table 1:** Identification of problems and potential solutions (Authors).

Issue	Potential Solutions	Who Wrote about It
1. Consistent definition of groups creation	- Adequate number of groups (see French, German, or Dutch examples in the text) - The number of classification levels (classification based on a cumulative score of secondary diagnoses provides a more accurate definition of case severity)	[5,10,11,12,13,16,17]
2. Tendency to increase the seriousness of cases	- Higher number of classification factors (e.g., using patient age as an element for group classification in some situations)	[14,15]
3. Focus on quality	- Higher weight to quality indicators in the reimbursement system to set clear rules and procedures for quality	[10,16,17]
4. Base rate convergence	- To unify rates at the national level through a regulation corridor	[18]
5. Repeated hospitalisation and length of stay	- Not paying for the subsequent hospitalisation if it occurs within a certain period of time after the patient is discharged; - Paying more for shorter hospitalisations, however, above a certain number of treatment days the case must be coded under a greater severity level	[19]
6. Professional coding	- Creation of a coder manual	[20]
7. Cost billing system	- Method of costing based on ABC calculation. Similar to the bottom-up micro-costing allocation method, this method assesses medical services based on detailed cost components of individual cases, taking into account centres of highly specialised care (higher cost due to depreciation of expensive technology, higher wages, etc.).	[12]
8. Level of centralisation	- To give healthcare providers greater autonomy - To manage reimbursement at the national level to a lesser degree	[12]

Source: Authors.

### 2.2. DRGs and Motivation of Hospitals

DRGs can be perceived as some kind of compromise between the fee-for-service and lump-sum payments [21]. As such, the system is expected to have an important impact on the behaviour of hospitals and their efficiency parameters. Based on the literature, the three main issues connected with the DRG-based payment systems’ influence on hospital behaviour are: (1) motivation to increase hospitals efficiency, (2) hospital benchmarking (comparison among and/or within hospitals, comparison with reference hospitals, etc.) and (3) source of information for hospital managers (monitoring concrete activities which may help managers to identify problems and their potential solutions) [4,21,22].

Among these issues, the motivation to achieve efficient behaviour might be seen as the most controversial issue (see more in [22]). In theory, a DRG should motivate the hospital to be more effective, i.e., to use its resources optimally, to maximise revenues and to reduce costs. However, based on Quentin et al., the effects of DRG on efficiency have been highly controversial (reducing cost without the quality improvements) [22].

There are three main incentives for hospitals resulting from DRG-based hospital payment systems that have (both intended and unintended) consequences on efficiency, quality and technological innovation: to reduce costs per treated patient, to increase revenues per patient and to increase the number of patients [22]. All hospitals, including the most efficient ones, are incentivised to continually reduce costs (e.g., by reducing the length of stay; by avoiding duplicity of and unnecessary tests; by replacing costly treatments by similarly effective but less costly alternatives, etc.). However, unfortunately, if the incentives for cost reduction are too strong and if regulatory authorities do not have sufficient capacity to adequately monitor the quality of care, DRG-based hospital payment can lead to cost reductions without any improvements in efficiency, e.g., inappropriately discharging patients or service intensity reduction to a level at which necessary services are withheld from patients [22].

According to Simon, DRGs may be seen as a tool enabling the hospital to identify ineffective activities and procedures, with the aim of achieving the most satisfactory results while providing the minimum possible level of care [21].

Hospitals are likely to be over- or underpaid for specific DRGs if cost data are inaccurate [16]. Although profitable DRGs may, in practice, compensate for less profitable DRGs, hospitals are disincentivised to improve efficiency for certain groups of patients if cost accounting leads to overestimated payments for a specific DRG. On the other hand, hospitals are disincentivised to provide unprofitable high-quality care if cost accounting leads to underestimated payments for a specific DRG [16].

## 3. Background: Financing Health Service Providers in the Czech Republic

The Czech healthcare services, as well as doctors’ skills, generally rank well in international tables [23]. The Czech Republic has a system of statutory health insurance based on compulsory membership in one of the health insurance funds. The insurers are quasi-public, self-governing bodies that act as payers and purchasers of care. Czech residents may freely choose their health insurer as well as healthcare providers [24].

Health indicators, such as infant and maternal mortality and life expectancy, have continuously improved in the past two decades. Like any country, the healthcare system faces the challenges of rapidly expanding possibilities of medical treatment and research with restricted public resources (see e.g., [23,24] to obtain more information about the Czech Health system). Balancing between maintaining open access to healthcare and the need to improve efficiency has kept the country in constant debate about the future of its healthcare [23].

Several reimbursement methods in financing health service providers are used in the Czech Republic: fee-for-service, lump-sum payment, per-day payment and DRG payments. Services are reimbursed to healthcare providers retrospectively. The conditions of healthcare reimbursement are defined by law. In-patient care in the Czech Republic is divided into three groups: (i) intensive care, (ii) other hospital services such as aftercare treatment, rehabilitation etc., and (iii) day cases [8]. The in-patient care reimbursement is calculated using a complicated equation in which the DRG payments are partly used for the intensive care only (44% of intensive care in 2021 but only 7% in 2020).

Reimbursement for in-patient care on the basis of DRGs started to be taken seriously as early as the 1990s in the context of the transformation of the Czech healthcare system, despite heavy criticism from some experts [25]. A real DRG pre-development project in the Czech Republic was initiated in 2007 and hospital care was supposed to be partly financed based on a DRG as of the following year. However, this start was delayed one year to 2009.

The introduction of a DRG in 2007–2009 was mired by many problems. Numerous changes were put in place over time (e.g., code additions/changes, ‘economic outliers’ introduction, rate adjustments). However, problems persisted. According to Roegnerová, the hospital sample was too small and the relative weights were calculated based on just four hospitals out of 150 [26]. There were also issues with system homogeneity—both in time and place (in terms of hospitals involved in the pilot)—and, last but not least, healthcare providers’ perceived lack of predictive capabilities.

All this led to two decisions made in 2014. First, the decision to stop financing hospital care based on a DRG and to temporarily return to flat fees in 2015. Second, the decision to transfer the DRG agenda to the Institute of Health Information and Statistics of the Czech Republic (ÚZIS), an organisational unit of the Ministry of Health, despite the fact that ÚZIS is not authorised to make any changes that could affect reimbursement, i.e., it cannot impose specific reimbursement mechanisms. In 2015, ÚZIS set up a network of hospitals (covering four of five hospital categories recognised in the Czech Republic) that provide reference data used in the development of new methodological documents.

As a result, the only changes related to the original DRG system implemented in the Czech Republic since 2015 were those necessary for the continuous functioning of the system (for example, the grouper and definition manual have not been updated at all since 2015 [27]). To restart the DRG on the new and better basis, the DRG Restart programme was then launched in 2016 with the objective of ‘building a long-term, sustainable data, information and personnel base for the optimisation and continuous development of the hospital care payment system in the Czech Republic and increasing the predictive power and efficiency of payment mechanisms for this segment of healthcare’ [28]. According to Malý, this project, in its first stage, can serve as a good example of intelligent, evidence-based policymaking, as it has been designed in a ‘scientific’ way and is run and managed by persons with a strong and respectable record as scientists and experts in this specific field [1].

Collection of calibration data started in the same year and continued until 2018. Data were also sourced from health insurers and the National Register of Hospitalised Patients. In spring 2018, the approved CZ-DRG implementation concept was approved, including a schedule and methodological materials. The DRG Restart is expected to end in 2023 when the original DRG system is supposed to shut down and be fully replaced by CZ-DRG through specific legislative changes [29].

In light of the above, it is not surprising that the reimbursement mechanism currently used in the Czech Republic is considered unsustainable by top authors on the topic [30,31]. We summarise their core opinions below:

1. The biggest problem of the current DRG is the poor definition of groups, which is not uniform in clinical or economic terms. This means that the real cost does not correspond to the current diagnosis classification and cost is not predictable with sufficient accuracy. This lack of clarity probably stems from the unfortunate choice of the NRC reference networks and incomplete definition of cost involved in patient hospitalisation, as well as an absence of cost deviation analyses.
2. The inaccurate classification system resulted in incorrect reporting of performance delivered or contracting care from departments other than those where the patient was treated (e.g., reporting operating theatre treatment as treatment performed at hospital beds). This resulted in inequalities between reported treatment and the real cost and resources consumed. There are even cases where a diagnosis is impossible to code and properly report to insurers.
3. A frequently discussed topic is the discrepancy in base rates, which is due to ‘individual base rates’ (IZS). Rates can differ for different healthcare providers but the decree also allows different insurers to negotiate different rates. Base rates can be unified only in a situation where the system has a predictive capability of adequate quality.
4. Another problem stems from the diagnosis coding procedure used in different hospitals. It is not uncommon for doctors to do the coding themselves based on medical documentation. The coding might then be checked by a coder but it is impossible to check all coded cases due to time constraints. Coders are currently trained primarily through courses. It is, therefore, debatable whether doctors should be responsible for coding, especially at a time when hospitals are struggling with a lack of doctors.

## 4. Materials and Methods 

The goal of this paper is to study the effects of the current Czech DRG system on hospitals’ financing and, on this basis, to propose possible changes to the reimbursement mechanism in the Czech Republic. The core research question(s) is if the current DRG might be seen as fair and motivating.

In terms of methodology, we utilised as our main method the qualitative research method to achieve planned results. Certain quantitative research elements, appropriate for this study, are also included.

To illustrate how the reimbursement mechanism works in the Czech Republic, the authors evaluated the mechanism’s application in three selected regional healthcare establishments in the period of 2012–2018. The healthcare establishments were selected in the way that (1) they have the same legal form (allowance organisations of regions) and (2) they belong to the same four Czech DRG categories (this is the ‘regional hospitals providing comprehensive types of healthcare’ category which comprises 76 hospitals). All these hospitals were asked to participate in this study. Unfortunately, only three hospitals agreed to cooperate. The small size of the sample is one of the limits of this analysis.

The surgery and orthopaedic wards were chosen for our study, as these wards were comparable in terms of size, structure and extent of care provided. All three hospitals used a unified management IT system. Seven DRG bases that represent typical activities performed in the given wards were selected for the purpose of our case study:

1. appendix operations (0605),
2. laparotomy of groin, thigh, umbilical or epigastric hernia (0608),
3. laparoscopy of groin, thigh, umbilical or epigastric hernia (0606),
4. laparoscopic cholecystectomy (0704),
5. cholecystectomy, other than laparoscopic (0703),
6. total knee replacement—TEP (0818),
7. arthroscopy (0819).

The authors delivered their own quantitative cost and revenue analysis for the selected DRG bases, or rather an analysis of the revenues generated by each DRG base. The cost analysis included the cost of the specific wards as well as the cost of central operating theatres, in addition to the primary ward cost and the cost of auxiliary and infrastructure activities. The authors used the ABC method to calculate the costs (using primary data collected in hospitals), where the costs were assigned to five defined activities: admission (A1), anaesthesiology (A2), surgery (A3), hospitalisation (A4) and discharge (A5).

Cost coding was based on qualified estimates and specific equations, taking into account the intensity of labour necessary to carry out each activity. In this respect, a ‘people activity matrix’ had to be created in order to express the amount of time needed by staff to carry out each activity. In light of the information obtained, it was very complicated to set the average time that the staff need to devote to a patient during the hospitalisation stay, as this data were influenced by the bed occupancy and the severity of the given case. This is why, in the case of hospitalisation, only the personnel costs of the physicians and physiotherapists were allocated directly. The example for the total knee replacement base is shown in Table A1 (see Appendix A).

Cost identification and their assignment to each individual diagnosis were complicated by the fact that costs were allocated differently in the studied hospitals, and in some cases the impossibility of finding all the necessary information which did occur. The example of the cost matrix for the total knee replacement base (0818) in hospital B is shown in Table A2 (see Appendix A). Due to cost allocation in this hospital, the ‘surgery’ activity was divided into two partial activities: activity A 3.1 represents the cost of central operating theatres and activity A 3.2 represents the personnel and material costs assigned to the relevant ward.

To calculate the cost per activity unit, we had to set relational values for each activity (admission—number of patients; anaesthesiology—number of patients; surgery—length of surgery and number of patients; hospitalisation—number of days in care; and discharge—number of patients). In light of the data obtained, the cost object was defined as the average patient of the specific DRG base in the studied wards. As an example, Table A3 shows the situation in base 0818 (see Appendix A).

In the revenue analysis, we calculated the revenues per average patient in the base in the studied wards. The revenue was not calculated directly but was provided by the healthcare providers from the unified management IT system.

To obtain a direct opinion of the core stakeholders, we also conducted semi-structured interviews with the DRG experts in order to evaluate the reimbursement mechanism’s use in 2012–2018.

## 5. Results

### How DRG Actually Works in the Czech Republic (Case Study of Three Co-Operating Hospitals)

As described in the methodology, we performed a cost and revenue analysis for selected DRG bases in three co-operating regional hospitals. In this section, the results of the analysis are presented.

The comparison of the average cost and revenues per patient in the three hospitals for the selected bases can be seen in Table 2, Table 3, Table 4, Table 5, Table 6 and Table 7. The Case Mix Index (CMI)—a relative value assigned to measure the average severity level of hospital procedures—is shown in the third column of the table. The CMI specifies the financial intensity of cases in each base and representation of DRG groups. Table 2, Table 3, Table 4, Table 5, Table 6 and Table 7 indicate that there is no direct link between CMIs and payments to hospitals.

The summarised results (Table 2, Table 3, Table 4, Table 5, Table 6 and Table 7) show that there are DRG bases with a high degree of homogeneity among providers in terms of cost, an example is base 0608. At the same time, there are DRG bases with a marked differentiation such as 0818; this result might be seen as surprising as the total knee replacement base is usually known to be homogenous—this may be caused by the small size of the hospital sample.

The fact that the differences in costs are extremely high cannot be simply explained by the providers’ different levels of efficiency because the current system lacks clear standards that would define the required content of hospitalisation cases of intensive hospital care. The cost differences also stem from contracts on healthcare services and from the structure of each case in each ward. Higher personnel costs also play a role (higher percentage of physicians) as does a higher volume of requested care and higher cost of depreciation of modern medical technology. The differences in cost might also be influenced by the hospital cases in the given ward, the share of intensive and planned care, operating theatres working time, etc. [27].

Regarding revenues, the tables show that hospitals do not seem to receive similar reimbursement for the same diagnosis. The difference may have two sources, i.e., different case mix and different approaches of health insurance companies.

**Figure 1:**
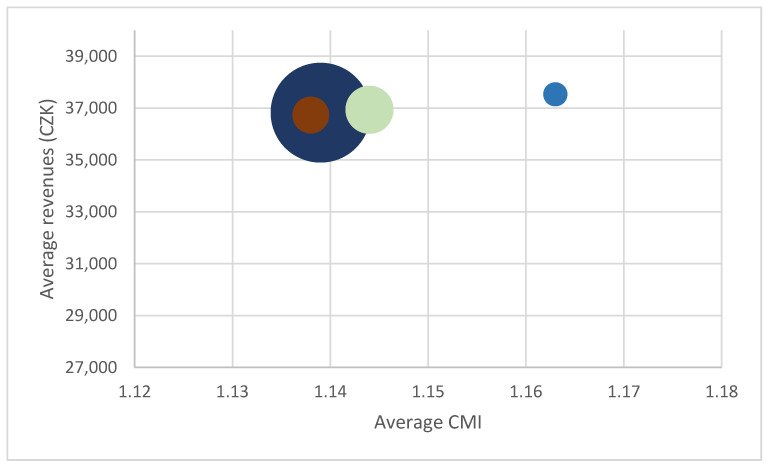
Relationship between revenues and CMI, base 0606, Hospital A.

**Figure 2:**
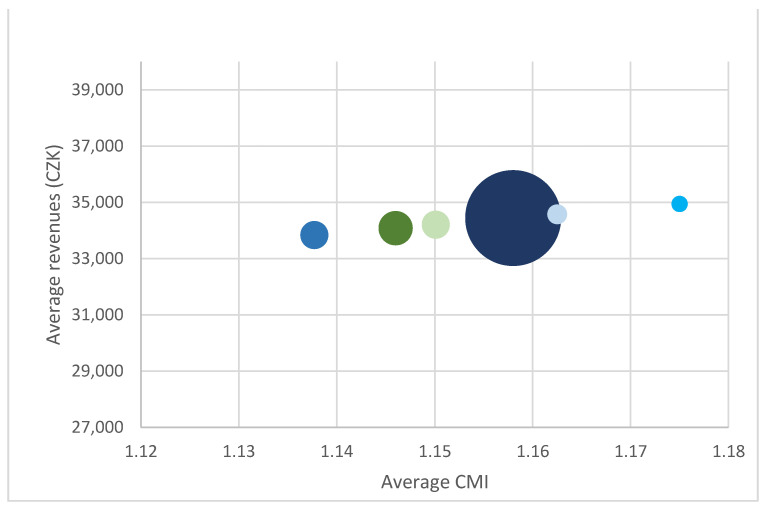
Relationship between revenues and CMI, base 0606, Hospital B.

**Figure 3:**
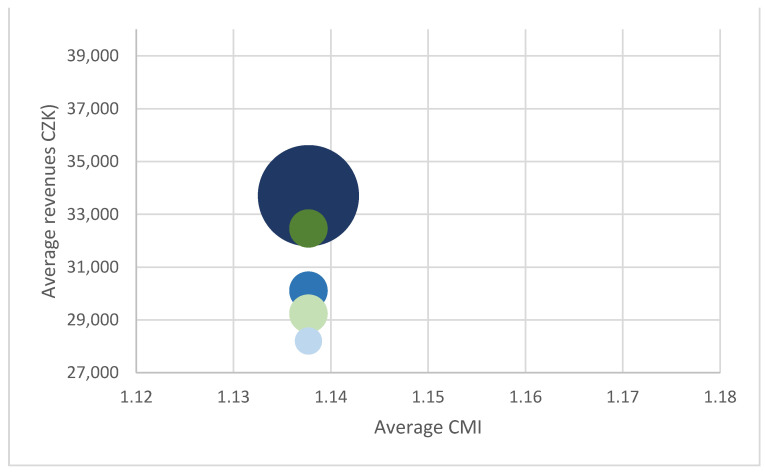
Relationship between revenues and CMI, base 0606, Hospital C.

It is interesting to observe the relationship between the average patient CMI and revenues from different insurers. As an example, the following three charts (Figure 1, Figure 2 and Figure 3) show the situation in case 0606 (laparoscopy of groin, thigh, umbilical or epigastric hernia). The charts illustrate the relationship between the revenue and CMI, and at the same time the different frequency of individual insurers is shown. The individual insurers are colour-coded and if the same amount of average revenue and CMI for more insurers appears, brown is used.

This comparison clearly shows rate differences in the given year not only amongst hospitals but also within hospitals. Different revenues from different payers indicate that although reimbursement depends on the CMI level, individual rates play a role as well. Our results indicate two (probably interrelated) problems, i.e., limited impact of the use of DRG reimbursement on the hospital funding and the limited fairness of the existing reimbursement system. Being aware of the small size of sample, both issues were discussed with expert opinions who confirmed their validity.

The ex-minister of health and the ex-director of VFN hospital, Dana Jurásková, claimed that ‘unfortunately, the Czech reimbursement mechanism is still too sophisticated and unpredictable. In this situation, it is very difficult for the management staff to plan the mix of care to provide in such a way that it would be sustainable or even beneficial for the hospital. In addition, the possibility to behave in this manner is strongly limited for the public hospitals in the Czech Republic. Moreover, the total costs per patient which is used in this analysis are usually not monitored in the Czech hospitals—changing this practice will be expensive and demanding. However, only when the management staff really knows all the costs of their hospital, can they adopt their strategy and their behaviour in order to “fit” into reimbursement’. She added that ‘historically, the efforts to introduce DRG as the main healthcare reimbursement mechanism in the Czech Republic faced fundamental obstacles. One of which is the heterogeneity of DRG cases that were taken over from other countries. That was also the reason—and not the only one—to start the cultivation process, within the so called DRG Restart project. I perceive this as a very good decision’.

Jan Kvaček, director of Bulovka Hospital, agreed with this when he described the DRG Restart project as a project showing who is efficient and who is not. He claims that ‘his hospital is, in most cases, deeply under the reference average—so, cost efficient and at the same time, his hospital is in the red. How is it possible? Normally in the market, one who has the lowest costs is someone who beats others. Why is it not like this in the healthcare sector? It is because hospitals are receiving historically set payments—for the same CMI, they are receiving different money. And the differences are huge: someone receives 20 while others receive 65 …’ [32].

The same problem was mentioned by Michal Čarvaš, the South-bohemian hospital Executive board member who claimed that ‘today we can see significant differences in revenues for patients in the same hospital chamber—depending on the insurer, the revenues differ about 30–40%. And I am not even mentioning that these differences depend on the type of health establishment: department, region or university hospital. The basic care in the university hospitals is overestimated and helps to finance the undervalued specialised care which is expensive and badly set in the reimbursement mechanism. The whole system is fundamentally deformed’.

## 6. Discussion

The data obtained by our analysis documents several critical problems of the current DRG system in the Czech Republic. The results of our analysis indicate that revenues are greatly influenced by the individual base rate (rate negotiated with insurer). In correlation with the interviews, the data obtained in our analysis confirm that the largest differences between costs and revenues might be observed in the case of the largest of our co-operating hospital (overestimated care due to the historically set reimbursement mechanisms). The results of our study show that although base rate convergence for providers is expected to be the next logical step, it might be very unfavourable for some healthcare providers given the current reimbursement setting. Defining a minimum base rate should not significantly impact any of the studied hospitals, therefore an overvaluation of the convergence corridor is unlikely. Efforts to avoid a deepening of the difference in individual base rates are translated in the current reimbursement decree. Our calculations indicate that a sudden convergence of base rates, e.g., through an average base rate for all hospitals, would result in a more sizeable compensation for costs in Hospital C and for orthopaedic patient cost in Hospital B. The latter provider would also see an increase in revenues from patients in surgery bases. The cost of patients in surgery bases in Hospital A, on the other hand, would be covered insufficiently.

The revenue analysis leads us also to the conclusion that providers whose average revenues grow in correlation with the growth of the average CMI have a more favourable reimbursement setting. A comparison of revenues from specific bases does not yield such clear-cut correlations. The differences are due to different rates negotiated by providers with different payers. Another factor is the volume of reported care compared with the reference period. This is a problem for efforts to erase reimbursement differences because current payments are tied to payments in previous years. Providers have little influence over the second factor, which takes some degree of responsibility from payers.

In terms of costs, our data show that the real cost does not correspond to the current diagnosis classification and, as shown from the interviews, cost is not predictable with sufficient accuracy.

Moreover, the information received from the interviews illustrates that the current DRG system is not motivating. Respondents were asked to comment on the consequences of various types of payment calculation used between 2012 and 2018 and their experience with and impression of reimbursement changes in this period. An interesting finding is that despite rather turbulent changes in the reimbursement policy taking place in 2012 (from 100% lump-sum payment to almost 100% DRG), hospital representatives did not describe 2012 as a major turning point in this area. The same is true for all the other years of the studied period. The hospitals even indicated that the changes to reimbursement decrees had little impact on their establishments. However, what the respondents were highly critical of was the instability of the system and artificial increases in wage tariffs by the Ministry of Health without reflecting these wage hikes in reimbursements. Personnel costs make up a considerable portion (around 50%) of the total cost incurred by regional hospitals [33].

This shows that the Czech reimbursement mechanism ‘lives a life of its own’ with little ties to reality.

Obviously, the delivered analysis has some limitations. First, the sample of hospitals is small, the authors analysed the situation in three out of 76 regional hospitals which provide a comprehensive type of healthcare services in the Czech Republic. This small size of the sample represents the biggest weakness of the analysis; therefore, the results of the analysis were consulted and confirmed with experts. Second, the variability of the selected hospitals is relatively low. It would be more than interesting to enlarge the sample, ideally with university hospitals where one can expect that the differences between cost and revenues will be expressive. There are some limitations stemming from the selected ABC method that is very demanding in data collection. As mentioned in the text, some data are imperfect. However, these limitations should not have any impact on the global picture received.

## 7. Conclusions

Our study (despite the small sample used) suggests that the current implementation of the DRG system in Czech reimbursements is set in a way that is very similar to traditional flat rates and is unlikely to yield major positive effects to the DRG mechanism, such as predictability of payments for hospitalisation cases, care quality and efficiency, and transparent financing. The results of our study and the opinions of experts indicate that despite frequent changes to reimbursement decrees and an effort to include DRG in intensive hospital care reimbursement, there is still a great need to further cultivate the system in the Czech Republic.

Based on our results and in compliance with the conducted interviews, we are convinced that a deep systemic change of the reimbursement mechanism in the Czech Republic is necessary. The main expected output of the DRG Restart project is to primarily include classification changes. Even if the improved classifications are indispensable, it cannot be seen as a real systemic change of the reimbursement system.

To reflect the general problems defined above, we propose five partial measures that are, in our eyes and in the eyes of experts, indispensable prerequisites facilitating the systemic change of the reimbursement mechanism in the Czech Republic that will lead to the cultivation of the Czech DRG:

1. the correct diagnosis classification setting and cost predictability,
2. availability of standardised, high-quality cost information and accessible hospital benchmarking,
3. the gradual base rate convergence,
4. less government dictation on the wage tariffs,
5. large number of costs-collecting hospitals.

As for the first measure, a higher quality of classification seems necessary for system improvement to benefit all participants. The case mix index should reflect the real costs. This means that the real cost must correspond to the current diagnosis classification and cost must be predictable with sufficient accuracy. The DRG Restart project is proposing to increase the exactness of classification through secondary diagnoses and their scores, even with a smaller impact on reimbursements. In the light of the results yielded by our study, this seems to be a reasonable solution. The new classification should give greater weight to the combination of the main diagnosis and reported procedures. However, it should not overlook the importance of secondary diagnoses either [33]. Another key condition is correct reporting of secondary diagnoses. We can look at the example of two of our analysed healthcare providers: while Hospital B has on average 2.6 secondary diagnoses reported per case in this base, Hospital C has only 0.62 secondary diagnoses per case on average. Even if the size of the studied sample is small, this difference is considerable as both hospitals belong to the same DRG category (regional hospitals providing comprehensive care). Although when comparing hospitals we must take into account representation of different severity groups without each base, the difference in reported care is very clear in this case and might be one of the reasons for the negative balance of calculated cost and revenues generated by this healthcare provider.

The next step in efforts to make the current classification more accurate should make sure that a patient’s age is reflected in the relative weight of care. In our calculations, we encountered a problem with personnel cost attribution to treatment days. This is a general trend which is valid regardless of the size of sample. One of the reasons for selecting an indirect attribution of personnel cost is the fact that hospital staff have declared widely different levels of patient care difficulty depending on the patient’s age, complications and co-morbidities. As personnel costs make up a considerable portion of the total cost incurred by healthcare providers, this seems to be a major failure of the current DRG system. The DRG Restart project classification promises to take patient age into account in determining the relative weight of a case. Patient age should compensate for potential disadvantages suffered by those providers who happen to treat more elderly patients than the average. Finance distribution by payers should then correlate more with the demographics of a population. These changes, which are crucial, will allow hospitals to make medium-term strategies and will lead to more open transparency of the reimbursement. This could therefore make the DRG more motivating.

As for the second measure (analytical evidence), it is crucial to unify the analytical evidence in all hospitals (not only in the reference hospitals). We believe that data for the hospital benchmarking should be accessible. The aim is to monitor the tendencies of hospitals to increase their efficiency (cost reduction and quality increase). It is necessary to emphasise the correct care and cost reporting. For the correct care reporting, this is reflected in the DRG Restart project which takes the standard medical care reporting as its basis. As a result, data can be collected not only from healthcare providers but also from insurers [34].

Conversely, the cost reporting is not treated by the DRG Restart. However, based on [21], the fairness of DRG-based hospital payment systems and the ability of these systems to encourage efficiency are to a large extent determined by the quality of the hospital cost information used to develop these systems and to calculate DRG weights. Unfortunately, suggested changes to the Czech DRG are mostly based on a more detailed specification and classification of hospitalisation cases (such as DRG categories introduction, changes in secondary diagnosis coding and five levels of severity). Changes to the reimbursement mechanism are not planned.

The third measure (base rate convergence) is related to the fact that, as shown in our analysis, there exist important differences in a hospital’s revenues per unit of production among providers and where the payments for the same case vary with different insurers—this general trend was confirmed in our analysis too. A gradual convergence of base rates could be a good starting point for a reduction of the effect of past reimbursements and deals with insurers. The DRG Restart project aims at creating the base rates map and to simulate the consequences of its changes. An accurate rate convergence should not increase the insurers’ risk of unreasonable hikes in reimbursements while increasing system transparency and possibly improving assessment of care efficiency. However, all these options require a sufficient and accurate classification because even this study confirms that the level of CMI, or of relative weights, is not in a way that would correlate at the given level with the cost of the relevant DRG base and the diagnoses included therein.

The fourth measure is connected to the problem of artificial increases in wage tariffs by the Ministry of Health without reflecting these wage hikes in reimbursements. Personnel costs make up a considerable portion (around 50%) of the total cost incurred by regional hospitals [33], and this seems to be a major failure of the current DRG. The annual index of the growth in personnel costs is higher than the growth in the revenues from the insurers. As it is a purely political decision, this might not be solved by the DRG Restart project.

The fifth measure (a large number of costs-collecting hospitals) is connected with our belief that the DRG quality is connected with the quality of initial data. Therefore, the aim of the DRG policy should be in increasing the number of hospitals that are voluntarily providing standardised data.

The final finding is the conclusion that the current DRG Restart project seems to aim at correcting the current problems of the Czech DRG, however the quality of proposed changes is limited and the impact of these changes on the behaviour of care providers is debatable, especially due to the effect of existing regulatory restrictions (for more about problematic regulatory environment see, e.g., [35]. Transparent and effective reimbursement is not just a matter of a scientifically advanced approach. To ensure that hospital care reimbursement really promotes improvements in care quality and efficiency of financing, truly systemic changes are also necessary.

## Figures and Tables

**Table 2:** Comparison of the average cost and revenues per patient, Hospital A.

DRG Base	Costs (CZK)	Revenues (CZK)	Difference (CZK)	CMI
0605	31,125	33,647	2522	1.042
0606	41,556	36,863	−4693	1.141
0608	21,930	22,276	346	0.690
0703	63,126	62,413	−713	1.933
0704	41,928	39,853	−2075	1.234
0818	85,597	108,604	23,007	3.491
0819	9011	19,620	10,609	0.608

CMI: The Case Mix Index.

**Table 3:** Comparison of the average cost and revenues per patient, Hospital B.

DRG Base	Costs (CZK)	Revenues (CZK)	Difference (CZK)	CMI
0605	30,044	29,728	−316	1.000
0606	29,966	34,369	4403	1.156
0608	22,262	22,212	−50	0.747
0703	70,199	69,126	−1073	2.324
0704	35,722	38,399	2677	1.291
0818	126,541	104,568	−21,973	3.516
0819	12,328	18,167	5839	0.611

**Table 4:** Comparison of the average cost and revenues per patient, Hospital C.

DRG Base	Costs (CZK)	Revenues (CZK)	Difference (CZK)	CMI
0605	36,083	29,678	−6405	1.057
0606	35,673	32,557	−3116	1.138
0608	20,774	20,442	−332	0.703
0703	66,537	57,639	−8898	1.961
0704	35,907	35,859	−48	1.234
0818	142,750	101,381	−41,369	3.493
0819	13,809	17,525	3716	0.604

**Table 5:** Comparison of hospitals: difference between costs for defined DRG bases.

DRG	Hospital A	Hospital B	Hospital C	Remark
0605	31,125	30,044	36,083	Homogeneous
0606	41,556	29,966	35,673	Too big a difference
0608	21,930	22,262	20,774	Homogeneous
0703	63,126	70,199	66,537	Almost homogeneous
0704	41,928	35,722	35,907	Almost homogeneous
0818	85,597	126,541	142,750	Too big a difference
0819	9011	12,328	13,809	Too big a difference

**Table 6:** Comparison of hospitals: difference between revenues for defined DRG bases.

DRG	Hospital A	Hospital B	Hospital C	Remark
0605	33,647	29,728	29,678	Differences not explainable by CMI
0606	36,863	34,369	32,557	Differences not explainable by CMI
0608	22,276	22,212	20,442	Differences not explainable by CMI
0703	62,413	69,126	57,639	Differences not explainable by CMI
0704	39,853	38,399	35,859	Differences not explainable by CMI
0818	108,604	104,568	101,381	Differences not explainable by CMI
0819	19,620	18,167	17,525	Differences not explainable by CMI

**Table 7:** Comparison of hospitals: difference between costs and revenues for defined DRG bases.

DRG	Hospital A	Hospital B	Hospital C	Remark
0605	2522	−316	−6405	Too big a difference
0606	−4693	4403	−3116	Too big a difference
0608	346	−50	−332	Too big a difference
0703	−713	−1073	−8898	Too big a difference
0704	−2075	2677	−48	Too big a difference
0818	23,007	−21,973	−41,369	Too big a difference
0819	10,609	5839	3716	Too big a difference

## Data Availability

The data that support the findings of this study are available from the corresponding author, upon reasonable request.

## Appendix A

**Table A1:** People activity matrix for the base 0818 (total knee replacement), Hospital C. The length of activities in minutes.

	A1	A2	A3	A4	A5
Physician orth.	20		108	10	20
Physician orth.			108		
Nurse orth.	20		108		20
Physician anaesth.		20	138		
Nurse anaesth.		20	138		
Nurse surg. theatre			108		
Enrolled nurse			108		
Physiotherapist	20			20	

Source: Authors.

**Table A2:** Cost matrix, base 0818, Hospital B (CZK).

Type of Cost	A1	A2	A3.1	A3.2	A4	A5
Material	1.331	0.571	286.132	234.981	831.619	5.435
Energy, water, gas	1.324	1.324	23.588	0.000	12.927	1.324
Reparations and maintenance	0.811	0.811	61.348	0.000	7.920	0.811
Travel costs	0.196	0.196	2.257	0.000	1.915	0.196
Services	5.688	5.688	102.236	0.000	55.551	5.688
Personnel costs	42.811	33.475	0.000	623.206	3017.898	33.475
Depreciation	0.179	0.179	121.209	0.000	24.282	0.179
Other costs	0.291	0.291	10.113	0.000	2.845	0.291
Aggregate activities	0.000	0.000	0.000	0.000	4.623	0.000
Sterilisation	0.000	0.000	313.116	0.000	4.729	0.000
Other internal costs	9.690	8.940	183.279	0.000	924.596	13.739

Source: Authors.

**Table A3:** Costs of average patient for the base 0818.

Orthopaedic Wards: Base 0818			
Activities	Cost [CZK]		
	Hospital A	Hospital B	Hospital C
Admission + anaesthesiology	845	933	1053
Surgery	8,701	16,078	10,481
Hospitalisation	27,282	38,941	57,530
Release	445	501	530
Separately billed material	48,324	70,088	73,156
Total	85,597	126,541	142,750

Source: Authors.

**Table A4:** Cost matrix, base 0605, Hospital A (thousands of CZK).

Type of Cost	A1	A2	A3.1	A3.2	A4	A5
Material	0.949	0.407	38.60	131.40	184.43	5.25
Energy, water, gas	2.265	2.265	4.978	0.000	12.824	2.265
Reparations and maintenance	1.099	1.099	8.537	0.000	6.224	1.099
Travel costs	0.288	0.288	0.177	0.000	1.633	0.288
Services	6.929	6.929	9.845	0.000	39.240	6.929
Personnel costs	26.856	26.856	0.000	210.849	1645.831	26.856
Depreciation	0.155	0.155	38.711	0.000	12.166	0.155
Other costs	5.050	5.050	10.779	0.000	28.597	5.050
Aggregate activities	0.000	0.000	0.000	0.000	37.133	0.000
Sterilisation	0.000	0.000	54.899	0.000	2.827	0.000
Other internal costs	12.748	12.334	42.163	0.000	217.176	16.024

Source: Authors.

**Table A5:** Cost matrix, base 0606, Hospital A (thousands of CZK).

Type of Cost	A1	A2	A3.1	A3.2	A4	A5
Material	0.763	0.327	52.775	478.748	136.266	4.223
Energy, water, gas	1.821	1.821	6.807	0.000	9.475	1.821
Reparations and maintenance	0.884	0.884	11.674	0.000	4.598	0.884
Travel costs	0.232	0.232	0.241	0.000	1.206	0.232
Services	5.574	5.574	13.461	0.000	28.992	5.574
Personnel costs	21.602	21.602	0.000	337.658	1340.950	21.602
Depreciation	0.125	0.125	52.934	0.000	8.989	0.125
Other costs	4.062	4.062	14.739	0.000	21.129	4.062
Aggregate activities	0.000	0.000	0.000	0.000	8.977	0.000
Sterilisation	0.000	0.000	75.068	0.000	2.089	0.000
Other internal costs	10.253	9.921	57.654	0.000	160.461	12.889

Source: Authors.

**Table A6:** Cost matrix, base 0608, Hospital A (thousands of CZK).

Type of Cost	A1	A2	A3.1	A3.2	A4	A5
Material	2.394	1.026	98.718	160.313	382.956	13.241
Energy, water, gas	5.711	5.711	12.733	0.000	26.628	5.711
Reparations and maintenance	2.772	2.772	21.836	0.000	12.923	2.772
Travel costs	0.727	0.727	0.452	0.000	3.390	0.727
Services	17.474	17.474	25.180	0.000	81.479	17.474
Personnel costs	67.724	67.724	0.000	542.459	2526.719	67.724
Depreciation	0.391	0.391	99.014	0.000	25.261	0.391
Other costs	12.735	12.735	27.569	0.000	59.381	12.735
Aggregate activities	0.000	0.000	0.000	0.000	16.601	0.000
Sterilisation	0.000	0.000	140.419	0.000	5.870	0.000
Other internal costs	32.146	31.104	107.844	0.000	450.951	40.409

Source: Authors.
